# 

**Published:** 1915-02-20

**Authors:** 


### CONTENTS.

PAGE

**Resident Appointments and the Present Emergency** ... ... ... ... ... ... 453

**What is "Ophthalmic Medicine"?** ... ... 454

**Hospital and Institutional News** ... ... ... 455

The Exchange of Doctor and Invalid Prisoners—Commission for a Hospital Secretary—Wounded Soldiers and the Epsom Spring Meeting—Personal Service for the Wounded—The Abolition of Outside Sick Visiting—Surgical Instruction Sixty Years Ago—The Post of Dispenser, Secretary, and Registrar—The Cardiff Society for Aiding Relatives—The Cottage Hospital Secretary—Bridge for Charity: The Subscribers' Point of View—A Missing Hospital Officer Found—Doctors' Fees for Examining Recruits—Honour for a Medical Superintendent—Should a Verandah Count as Floor Space?—The List of Lowest Prices—Building Schemes and Unemployment—The Special Hospital for Wounded Men—The War and the Coroners—The Maintenance of Military Patients—Medical Officer of Health and Military Duty—A Hospital for Permanently Disabled Belgians—Ireland's Provision for the Wounded—Useful Hints for Dispensers—Aspirin Substitutes—Condition of the Men at the Crystal Palace—This Week's Drug Market.

**Medico-Social Questions and the War** ... ... 461

The Profession and the Politicians.

**The Clinical Value of Poor-Law Infirmaries**... 462

The Root of the Medical Difficulty.

PAGE

**The Bombardment of Coast Towns** ... ... 463

Brighton's Medical Emergency Scheme.

**Leicester as a Hospital Town** ... ... ... 464

The Problem of Extended Accommodation.

**A Medical Causerie** ... ... ... ... ... 465

**Round the World** ... ... ... ... ... 466

Fellow-Workers and the Roll of Honour.

**The Evolution of the Modern Hospital** ... 467

I.—The Internal Revolution.

**The Pharmaceutical Society** ... ... ... 468

**The Editor's Letter-Box** ... ... ... ... 469

The American Women's War Hospital—The Clinical Value of Poor-Law Infirmaries—The Paddington Infirmary System—The Telephone in Medical Practice.

**Ancoats Hospital and the War** ... ... ... 470

**The Values of Stocks at Given Times** ... 471

King Edward's Hospital Fund for London Statistics.—Fifth Notice.

**Central Poor-Law Conference** ... ... ... 472

A Township for the Blind.

**The King at Cambridge** ... ... ... ... 473

**IV.—Some Provincial Hospital Chapels** ... 474

Royal Infirmary, Liverpool.

**St. George's Hospital**... ... ... ... ... 476

**The Institutional Worker** ... ... (Suppl.) 1

Answers to January Question: Receiving Goods Without a Steward.

The Importance of Weighing.

A System in Use.

